# Promoting agency and communication through powered mobility: a single-group, repeated measures intervention using the explorer mini in toddlers with motor disabilities

**DOI:** 10.3389/fresc.2025.1726259

**Published:** 2026-01-12

**Authors:** Liesbeth Gijbels, K. A. Ingraham, N. L. Zaino, M. E. Hoffman, J. C. Mizrahi, A. N. Sinclair, B. Y. Woo, A. Fragomeni, A. N. Meltzoff, K. M. Steele, H. A. Feldner, P. K. Kuhl

**Affiliations:** 1 Department of Speech & Hearing Sciences, University of Washington, Seattle, WA, United States; 2 Institute for Learning & Brain Sciences (I-LABS), University of Washington, Seattle, WA, United States; 3 Center for Research and Education on Accessible Technology and Experiences (CREATE), University of Washington, Seattle, WA, United States; 4 Department of Electrical and Computer Engineering, University of Washington, Seattle, WA, United States; 5 Department of Mechanical Engineering, University of Washington, Seattle, WA, United States; 6 Institute on Human Development and Disability, University of Washington, Seattle, WA, United States; 7 Department of Psychology, University of Washington, Seattle, WA, United States; 8 Department of Rehabilitation Medicine, Division of Physical Therapy, University of Washington, Seattle, WA, United States

**Keywords:** agency, communication, language development, motor impairments, powered mobility

## Abstract

**Background:**

Independent mobility in early life catalyzes development across motor, perceptual, cognitive, social, and linguistic domains. For young children with motor disabilities, however, opportunities to explore, interact, and learn from their surroundings are commonly restricted, which may limit developmental progress.

**Objective:**

To examine the effects of a play-based powered mobility intervention, using the Permobil Explorer Mini, on developmental and communicative outcomes in toddlers with motor disabilities, using a single-group, repeated measures design.

**Methods:**

Ten children with motor disabilities (ages 12–36 months) participated in a 12-session intervention in an enriched laboratory environment. The intervention consisted of repeated, play-based sessions using the Explorer Mini, an FDA-cleared powered mobility device. Developmental skills were evaluated pre- and post-intervention using standardized assessments, and language environments were systematically analyzed both in the laboratory and at home.

**Results:**

At baseline, participants scored significantly below age-matched norms across all domains (gross and fine motor, receptive and expressive language, cognitive, adaptive behavior, and social-emotional). Following the intervention, children showed significant raw score gains in cognition, receptive language, fine motor, adaptive behavior, and social-emotional development. Scaled score improvements in cognition and receptive vocabulary indicated growth exceeding expected age-related progression. Analyses of the language environment revealed significant increases in children's expressive vocalizations. Specifically, more frequent joyful (“delight”) vocalizations and fewer expressions of distress. Increases in delight vocalizations were further strongly correlated with receptive language gains.

**Conclusion:**

These findings suggest that introducing powered mobility during the toddler period can foster agency, exploration, and communication, catalyzing developmental growth beyond motor function alone. By providing young children with the means to move independently, powered mobility may open new pathways for participation, learning, and connection, laying critical groundwork for more equitable developmental opportunities in early childhood.

## 1 Introduction

Have you ever witnessed the delight of a toddler's new ways to explore the world, such as taking their first steps or learning how to crawl? These moments are more than a physical milestone, they are gateways to a world of exploration, independent agency, and discovery. As infants gain self-initiated mobility, their environment affords more opportunities to engage, learn, and connect. This ability to independently explore one's surroundings through crawling or walking, sparks cascading changes in motor, perceptual, cognitive, social, and linguistic domains [e.g., (1, 2)]. These changes are triggered not only by movement itself, but also by the learning provided by agentive control of one's own exploration (3, 4). Such engagement with the environment generates opportunities for interaction, problem-solving, and skill acquisition (5). For language specifically, advances in motor skills are accompanied by shifts in the content and timing of the language infants hear, highlighting critical connections between objects, actions, and the sound patterns (i.e., words) that represent them (6).

However, when these first milestones are impacted by a motor disability, the developmental trajectory can be markedly different. Infants and toddlers with motor disabilities often rely on caregivers for mobility, influencing how and when they encounter experiences that support development. These delays in achieving self-directed mobility might have downstream consequences across a broad swath of developmental attainments during the first years of life and beyond. Powered mobility technologies have emerged as an important tool to help these children. By enabling young children with disabilities to move through their environment independently, these devices promote not only physical movement but also social engagement, cognitive development, communication skills, and increased participation in daily life across home, school, and community contexts (7–10).

The Permobil Explorer Mini (11) is the first commercially available FDA cleared powered mobility device in the United States that is specifically designed for toddlers (ages 12–36 months). The device addresses many barriers experienced by other powered mobility devices, like difficulties with manual steering, restricted control options, low maneuverability, and noise (12–14). By reducing the complexity of operation and increasing accessibility, the Explorer Mini opens new possibilities for young children with motor disabilities to explore their environment independently, engage with caregivers and peers, and participate in meaningful early social interactions (15, 16).

Despite these advances, a critical knowledge gap remains. It is not yet well understood how independent exploration via toddler-specific powered mobility devices influences broader developmental outcomes, particularly during the sensitive early years. While the Explorer Mini represents a significant technological advance, more research is needed to determine whether and how access to self-initiated movement through such devices can catalyze cascading developmental gains, similar to those observed in typically developing children following the onset of crawling or walking (5).

The current study addresses this gap by examining how guided use of the Explorer Mini shapes early learning and communication in toddlers with motor disabilities. Specifically, we investigated whether access to self-initiated mobility and the development of a sense of agency via guided learning and powered mobility would lead to significant improvements in receptive and expressive language skills, as well as other developmental domains, including cognition, gross and fine motor abilities, and social-emotional development. Ten young children participated in 12 in-lab intervention sessions, during which their learning and engagement with powered mobility devices were monitored over time.

The present work aims to provide direct and meaningful support to young children and families in our local disability community, while also generating critical empirical evidence to inform early intervention practices. By demonstrating how accessible technology can ignite developmental progress, it offers a model for promoting equitable opportunities from the very start. We hypothesized that guided access to self-initiated mobility would catalyze developmental gains across multiple domains, with primary outcomes focused on language, cognition, and social-emotional development.

## 2 Methods

### 2.1 Study design

This study used a single-group, repeated measures intervention design to evaluate the impact of a powered mobility intervention in young children with motor disabilities.

Participants received a play-based powered mobility intervention program using the Permobil Explorer Mini, an FDA-cleared powered mobility device designed specifically for toddlers. All child participants completed pre-test and post-test sessions in the laboratory, scheduled one to two weeks before and one to three weeks after the intervention sessions. Participants were scheduled to complete 12 intervention sessions in the lab, held once or twice weekly over a period of 6–12 weeks. Each session lasted approximately one hour and involved the child, at least one caregiver, and a minimum of two researchers.

Sessions began with setup and sensor application, followed by two 15–20 min driving blocks separated by a 5–10 min break. Although the target drive and play duration for each session was 15–20 min, this was adjusted based on the child's engagement, temperament, and tolerance.

### 2.2 Participants

#### 2.2.1 Inclusion criteria

The inclusion criteria for the study were that participants: (a) were between 12 and 36 months of age at enrollment, (b) had a disability or developmental delay affecting movement, and (c) were able to tolerate sitting upright (with support) while moving through space for 15 min. Two other constraints were that the caregivers had to be willing to bring the child participant to the University of Washington for 12 in-lab visits and that these caregivers had to be proficient in English to receive instructions, complete forms, and so on.

#### 2.2.2 Recruitment

The child participants were recruited by the research team through multiple mechanisms: (a) social media postings, (b) emails to known rehabilitation professionals in the area, (c) fliers distributed at local healthcare clinics and hospitals, (d) the University of Washington Communication Studies Infant and Child Participant Pool, and (e) word-of-mouth recruitment efforts by our lab.

#### 2.2.3 Study sample

A total of 10 children with motor disabilities, accompanied by at least one caregiver, participated in this study (Table 1). At the time of enrollment, the children (8 boys and 2 girls) had a mean age of 21.8 months (*SD* = 5.78). Mobility levels at the time of enrollment were characterized as follows: Three children were non-mobile, two were able to sit with support, four were at the rolling stage, and one child was capable of cruising. In terms of expressive language development, seven children were prelinguistic, one child had reached the stage of babbling, one child produced their first words, and one child expressed two-word sentences at the start of the study. Of the seven prelinguistic children, one (P9) was not able to produce vocal sounds because of a breathing tube, and four children had conditions related to muscle tone that affected their physical ability to produce speech (hypotonia [P2], cerebral palsy [P5, P6, P8]).

**Table 1:** Participant demographics and characteristics at study entry.

ID	Age (months)	Sex	Ethnicity	Disability Type	Mobility	(Expressive) Language Development
P1	31	F	White/Asian	Neurological	Non-mobile	Prelinguistic
P2	14	M	White	Neurological	Sitting	Babbling
P3	16	M	White	Orthopedic	Cruising	Two-word stage
P4	21	M	Preferred not to answer	Neurological	Sitting	Prelinguistic
P5	28	M	White	Cerebral Palsy	Rolling	Prelinguistic
P6	27	M	White/Asian	Cerebral Palsy	Non-mobile	Prelinguistic
P7	16	M	Mixed	Spinal Myopathy	Rolling	First words
P8	18	F	Black African	Cerebral Palsy	Rolling	Prelinguistic
P9	24	M	White	Orthopedic	Non-mobile	Prelinguistic
P10	23	M	White	Hypoxic Ischemic Encephalopathy	Rolling	Prelinguistic

#### 2.2.4 Consent

Caregivers of all participants provided informed consent for both their own and their child's participation, with optional consent granted for the use of their child's images in academic publications, under a protocol that was approved by the University of Washington's Institutional Review Board (#00,014,879). Participants were financially compensated with Tango Gift Cards: $25 per in-lab study visit, $10 per language questionnaire, and $25 per day of at-home language audio recording.

### 2.3 Protocol

#### 2.3.1 Room setup

To support the intervention, an open, enriched play space was created in the lab, allowing children to explore and control their mobility while interacting with the Explorer Mini (11), their caregivers, a wide variety of sensory and motor-based toys, and members of the research team.

**Figure 1:**
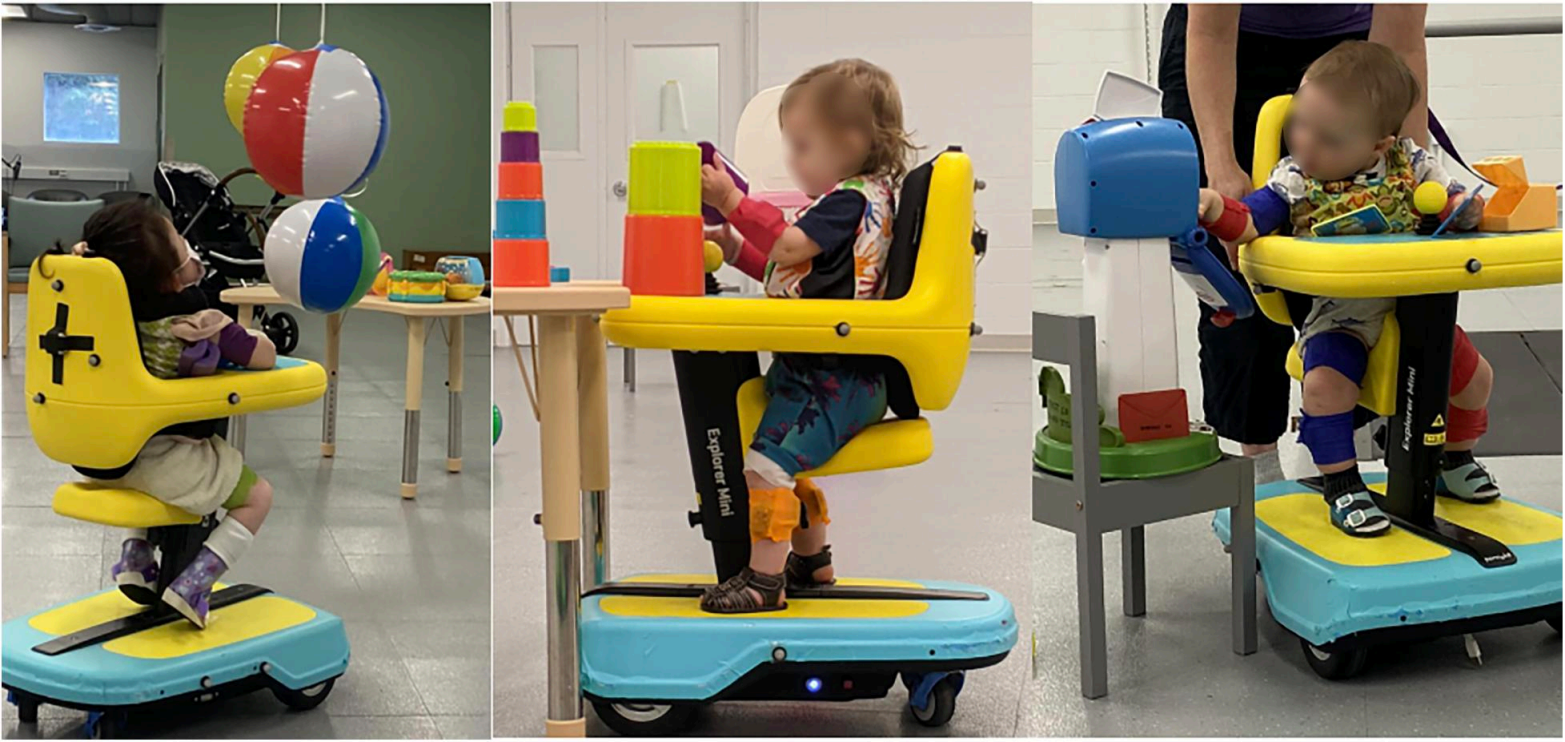
Play sessions were held within an enriched environment containing interactive toys arranged at eye level and within reach.

The play environment included a wide variety of toys, such as stacking cups, fishing rods, musical toys, switch-adapted electronic toys, and both digital and non-digital visual displays (e.g., disco lights, beach balls hung from the ceiling). These were arranged at the child's eye level and within reach (Figure 1). Toys were placed on tables and spread around the room to encourage exploration. Toys were customized for each visit based on caregiver-reported interests and the child's observed preferences.

#### 2.3.2 Powered mobility device

Participants used the Permobil Explorer Mini (11), the only FDA-cleared pediatric powered mobility device commercially available in the United States for children ages 12–36 months. For this study, an instrumented version of the Explorer Mini was developed in collaboration with LUCI's Sandbox program (17) to capture data on joystick interactions, wheel rotations, and body weight distribution during use [see (18), for further details]. Other technical data collection, although not discussed in this work, included use of the lab's optical motion capture system to record and analyze each child's driving paths, surface electromyography to record lower limb muscle activation in seated and standing driving positions, accelerometry data captured by inertial measurement units to quantify upper limb and trunk positioning, and the custom sensing capabilities of the in-lab Explorer Mini [see (19), for further details].

**Figure 2:**
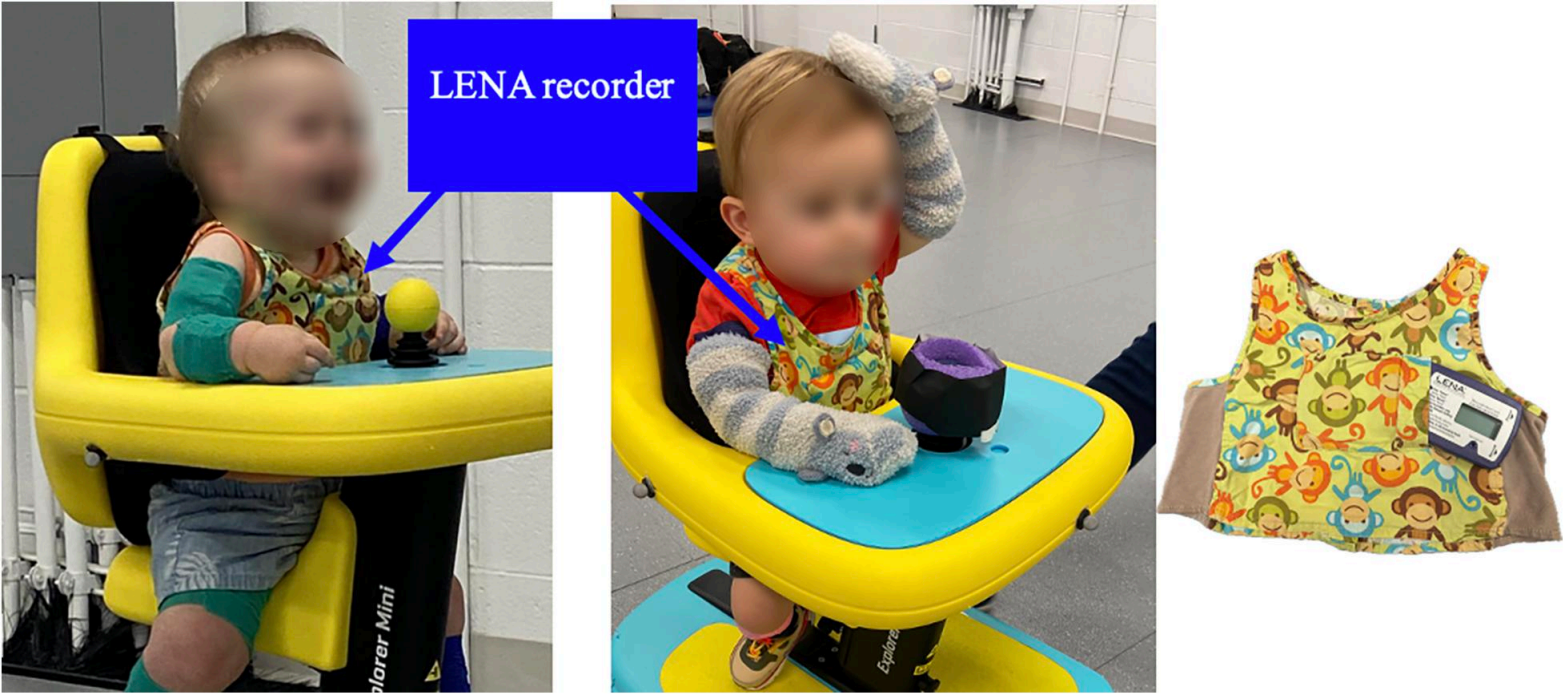
The children wore vests with a LENA recorder during each intervention session. The recorder did not interfere with the child's mobility and was able to capture all sounds from child, caregiver, researcher, and toys.

At the start of each play session, the Explorer Mini's seat and tray height were adjusted to ensure the child's comfort and joystick accessibility. The speed setting was customized based on the child's performance and preferences. Additional positioning supports, such as towels, foam, or pool noodles, were used as needed (Figure 2, right panel).

#### 2.3.3 Session guidelines

During each session, participants engaged in child-led, exploratory play in this enriched environment, with interactions guided by the Assessment of Learning Powered Mobility (ALP) tool and facilitating strategies (20), as well as *A Guideline for Introducing Powered Mobility to Infants and Toddlers* (21). Driving and play activities were customized to align with each child's individual learning stage. For instance, a child in the novice learning stage (ALP Phase 1) might explore the joystick with unintentional activation, show frustration, or express a desire to leave the device. Training at this stage focused on cause-and-effect play activities to draw attention to the joystick, hand-over-hand guidance to demonstrate that joystick activation moves the Explorer Mini, and engaging in-device games to build tolerance for using the device. In contrast, training for a child in the advanced beginner stage (ALP Phase 4) emphasized more goal-directed driving activities, such as playing hide-and-seek with a caregiver or toy. Caregivers were actively involved in each session and encouraged to engage with their child and the research team throughout the driving and play activities. Caregivers were encouraged to interact and play with their children as they wished. As a result, children were not required to access toys entirely on their own; caregivers could choose to engage verbally or physically and were free to move toys both within the room and on the child's tray.

The Assessment of Learning Powered Mobility (ALP) was employed not as a primary analytic tool, but rather as a means to confirm that each child achieved self-directed movement during the intervention. Specifically, reaching at least Stage 3 on the ALP, which all children did at minimum by the end of the intervention, served to validate our observation that participants were capable of intentional, self-initiated mobility. Detailed ALP scores are not reported, as the focus of this manuscript is on broader developmental outcomes.

### 2.4 Assessment instruments

#### 2.4.1 Bayley scales of infant development (BSID-IV): pre-/post-test

To quantify participants' developmental levels, a qualified pediatric physical therapist administered the *Bayley Scales of Infant Development, Fourth* Edition [BSID-IV; (22). This is a validated, norm-referenced set of tests designed to assess developmental domains from 1 to 42 months of age. The BSID-IV includes assessments of cognitive, language (receptive and expressive communication), motor (fine and gross motor), social-emotional, and adaptive subscales. All scales were assessed in the pre-test and post-test session. Administration of this scale took approximately 30–70 min. The BSID-IV has been validated for our study population (22), and both raw and scaled scores were used in data interpretation.

#### 2.4.2 Language ENvironment analysis (LENA)

##### 2.4.2.1 Pre-/post-test

One weekend before and one weekend after the intervention period, caregivers were asked to have their child wear a digital audio recorder, the *Language ENvironment Analysis* system [LENA™ Pro Version 3.4.0, (23)], and complete a daily activity diary to collect data about the child's at-home language and audio environment. These recordings provide a first-person perspective on the auditory worlds of the participants and record their utterances in their environments. The LENA device is a small, credit-card-sized language monitor placed in a vest and worn by the child (Figure 2) for a full day (approximately 10 h) on two consecutive weekend days. The recordings were analyzed both manually by a researcher and automatically by the program. To analyze these two-day recordings, the LENA program selected 100 30 s snippets (50 per day) for each pre- and post-test recording. We followed the snippet-selection procedure described in detail within the supplemental materials of Ferjan Ramírez et al. (24, 25).

The LENA system includes an automatic analysis method; however previous work from our lab has shown that not all automatic analyses are accurate (24). Given the complexity of the current population, we relied solely on manually scored data. For all 200 30 s snippets per participant, a highly trained research assistant scored the following variables: (a) number of snippets containing speech directed to the child, (b) number of snippets containing parentese speech (26, 27), (c) number of snippets containing standard speech (28), and (d) number of snippets containing speech directed to the child with one adult, and with more than one adult. This scoring was documented for all speech utterances, but also broken down by standard speech and parentese. We also recorded (a) total conversational turns in snippets, (b) mean conversational turns across snippets, (c) total number of snippets containing babbling, (d) total number of snippets containing babbling from the child when no speech was directed to the child, and (e) total number of words produced by the child. More details about these variables and their definitions can be found in Ferjan Ramírez et al. (25) and Ferjan Ramírez (29).

##### 2.4.2.2 Intervention sessions

During each intervention session, the child wore a vest with a LENA recorder (Figure 2) to capture the child's social engagement, their interactions with caregivers and researchers, interactions between caregivers and researchers, and other auditory events.

Sessions 1, 4, 8, and 11 for all participants were selected to be coded and transcribed by a trained research assistant in random order. A total of 34 30 s snippets per play session were randomly selected (15 during the first driving block, 4 during the break, and 15 during the second driving block). For these 34 snippets the following variables were scored: (a) percentage of snippets in which the child babbled, (b) total number of vocalizations across all snippets of a session, categorized by three types of vocalization [delight, displeasure, neutral (30–32), (c) percentage of snippets in which the child produced words, (d) total number of words produced across all snippets of a session, (e) percentage of snippets in which the child combined words, (f) longest utterance produced across all snippets, and (g) total number of conversational turns in 34 snippets. From adult speech, we counted: (a) percentage of snippets in which either a caregiver or a researcher was talking directly to child (calculated separately for each caregiver, for both caregivers combined when both were present, and for all adults combined), (b) percentage of snippets containing parentese, (c) percentage of snippets containing standard speech, (d) average number of parentese utterances in snippets in which any parentese was produced, and (e) average number of standard speech utterances in snippets in which any parentese was produced.

### 2.5 Analysis plan

The primary outcomes of this study were changes in developmental scores as measured by the Bayley Scales of Infant Development, Fourth Edition (BSID-IV), including both raw and scaled scores across cognitive, language, motor, social-emotional, and adaptive behavior domains. In addition, changes in expressive language and vocalization patterns were assessed using the Language ENvironment Analysis (LENA) system, where we looked at both adult-to-child interaction and child language production (Methods section 2.4.2 for more details). For analysis purposes adults were grouped as caretakers, researchers, or both combined.

To address the study objectives, statistical analyses were conducted in several stages. Initially, descriptive statistics were calculated to summarize participant demographics and baseline assessment scores. Baseline comparisons were performed to evaluate how participants' pre-intervention BSID-IV scaled scores compared to normative data for typically developing children, using one-tailed t-tests for each developmental domain.

To assess the effects of the intervention, pre- and post-intervention scores for each outcome measure were compared within participants using one-tailed paired t-tests. This approach was applied to both BSID-IV raw and scaled scores, as well as to LENA-derived measures of expressive language, including babbling and word production.

Changes in vocalization patterns across the intervention period were further examined using linear mixed-effects models of data from the LENA recordings. To examine changes in vocal behavior over the course of the intervention, we analyzed two complementary sets of child vocalizations: (a) babble and word productions, and (b) non-babble vocalizations. We focused on coding non-babble vocalizations produced by the child, as our population had limited expressive vocabulary due to motor impairments or the presence of medical devices (e.g., breathing tubes). These vocalizations were categorized into three types. The first category, *delight*, included non-babble vocalizations that signified positive emotions, such as laughing, giggling, or other joyful sounds, occurring at any point during the segment. The second category, *displeasure*, captured non-babble vocalizations indicating negative emotions, such as crying, whining, fussing, or other sounds of distress. The third category, *neutral*, comprised non-babble vocalizations that were neither positive nor negative, such as high-pitched squeals involving falsetto or a highly tense maximal pitch register, low-pitched growls with a creaky-voiced quality, or effort-related grunts. This framework allowed us to explore a wider range of vocalizations, offering valuable insights into the children's communicative behaviors beyond traditional expressive language.

In these mixed-effects models, the number of babbles and word utterances produced by each child per session served as the dependent variable, with intervention session as a fixed effect and participant as a random effect. A similar modeling approach was used to analyze composite non-babble vocalizations, which were defined as the sum of delight and neutral vocalizations minus displeasure vocalizations.

Associations between changes in vocalization patterns and developmental gains were explored *post hoc* using Pearson correlation coefficients. Specifically, correlations were calculated between changes in LENA-derived vocalization measures and improvements in BSID-IV scaled scores. These analyses were not part of the original analysis plan and were conducted after initial inspection of the data.

All statistical analyses were performed using R (version 1.3.1093), with the lme4 (36), nlme, stats, and psych packages. Given the small and heterogeneous sample (*N* = 10), the statistical power to detect effects was limited, and the generalizability of the findings is constrained. The results should therefore be interpreted as preliminary and exploratory, providing foundational data to inform future, larger-scale studies.

### 2.6 Data collection details

Nine of the ten participants completed all 12 intervention sessions. Participant P3 completed 11 sessions due to the 12-week time constraint. All children completed the pre- and post-intervention BSID-IV and in-session LENA recordings. Eight out of ten children completed pre-test/post-test LENA recordings. Participant P1 did not consent for this procedure and participant P4's data was excluded as they came from a multilingual home. The English proficiency of the caregiver(s) was sufficient to participate in the study, however the majority of the interactions from the pre-test/post-test recordings were not in English.

## 3 Results

### 3.1 Bayley scales of infant development (BSID-IV)

Given that validation of the BSID-IV has been based primarily on typically developing children, age-equivalent scaled scores on children with developmental delays are expected to be consistently reduced. To accurately capture changes over the 12-week intervention, and because improvements in raw scores reflect real developmental progress, we report both scaled and raw scores (7, 33, 34).

To establish a baseline, we compared participants' pre-intervention BSID-IV scaled scores in each developmental domain (cognitive, communication, motor, adaptive behavior, social-emotional) to normative data for typically developing children. Mean scores indicated most children scored 1–2 SDs below the mean for typically developing peers. Specifically, mean scores were: cognitive (*M* = 3.00, *t* *=* −7.72, *p* < 0.001), receptive vocabulary (*M* = 4.90, *t* = −3.20, *p* = 0.011), expressive vocabulary (*M* = 4.10, *t* = −4.38, *p* = 0.002), fine motor (*M* = 3.60, *t* = −6.46, *p* < 0.001), gross motor (*M* = 1.50, *t* = −17.00, *p* < 0.001), adaptive behavior (*M* = 5.40, *t* = −3.83, *p* = 0.006), and social-emotional (*M* = 5.25, *t* = −3.06, *p* = 0.018).

To assess intervention effects, pre- and post-intervention BSID-IV raw and scaled scores were compared (Table 2). Significant improvements in raw scores were observed for cognitive, receptive vocabulary, fine motor, adaptive behavior, and social-emotional domains (*p* < 0.05). Scaled scores showed significant gains in cognition (*M* = 1.40, *t* = 2.26, *p* = 0.025) and receptive vocabulary (*M* = 0.90, *t* = 2.21, *p* = 0.027). Expressive vocabulary and gross motor scores improved but did not reach significance.

**Table 2:** BSID-IV raw and scaled score differences before and after intervention for each developmental domain: cognitive, communication, motor, adaptive behavior, and social-emotional. Mean improvement shows the change in scores (post-intervention minus pre-intervention), the 95% confidence interval for the question of whether the difference was > 0, *t* and *p* values are shown. Significance is indicated by *.

Scale	Mean improvement	95% CI	*t*-value	*p*-value
Raw Scores				
Cognitive	18.50	(6.74, ∞)	2.89	.009**
Receptive Vocabulary	8.20	(3.54, ∞)	3.22	.005**
Expressive Vocabulary	2.60	(−0.88, ∞)	1.37	.102
Fine Motor	7.00	(0.82, ∞)	2.07	.034*
Gross Motor	4.50	(−1.63, ∞)	1.35	.106
Adaptive Behavior	2.91	(0.15, ∞)	1.99	.043*
Social-Emotional	11.50	(4.59, ∞)	3.16	.008**
Scaled Scores				
Cognitive	1.40	(0.27, ∞)	2.26	.025*
Receptive Vocabulary	0.90	(0.15, ∞)	2.21	.027*
Expressive Vocabulary	−0.30	(−0.77, ∞)	−1.15	.861
Fine Motor	0.00	(−0.39, ∞)	0	.5
Gross Motor	0.20	(−0.17, −∞)	1	.172
Adaptive Behavior	0.13	(−0.61, −∞)	0.32	.379
Social-Emotional	−0.13	(−1.33, −∞)	−0.20	.575

### 3.2 Language ENvironment analysis (LENA)

#### 3.2.1 Pre-/post-test

**Figure 3:**
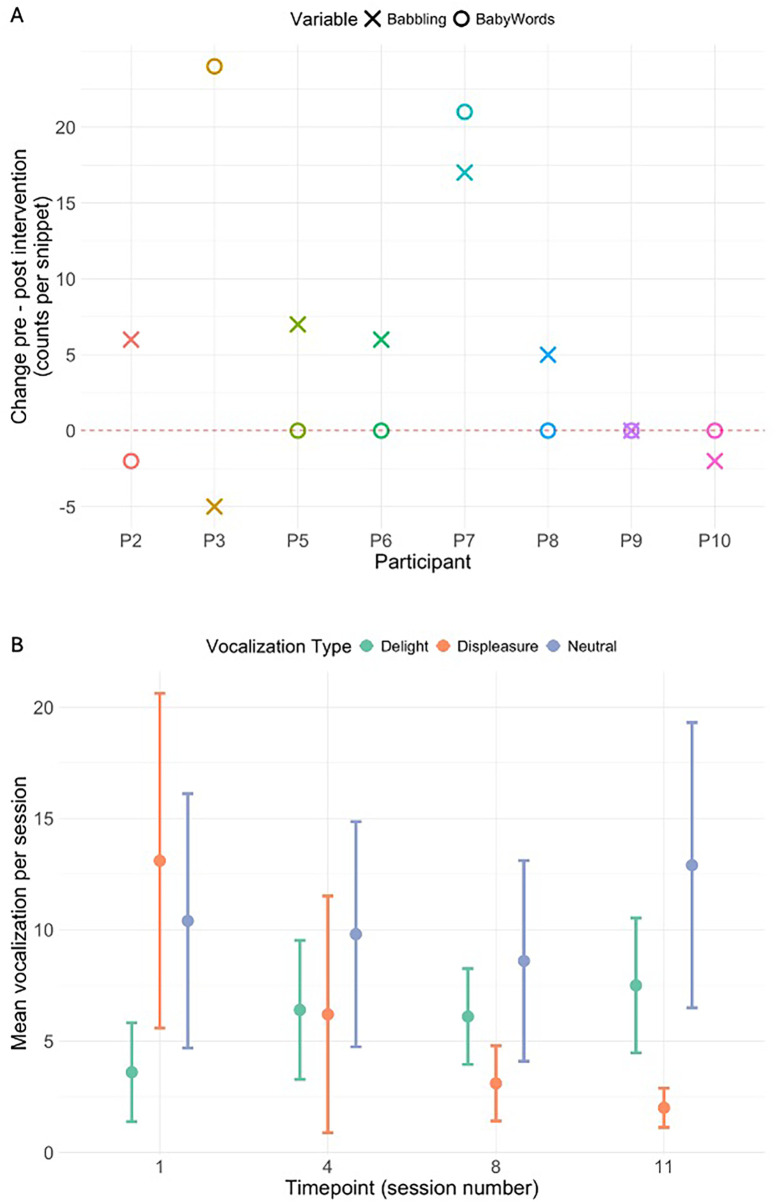
Panel **(A)** shows the change in counts of babble and words produced pre-test and post-test, per child participant. Counts of babble produced by the child are represented by X, whereas counts of words are represented by O. Panel **(B)** shows the average non-babble vocalizations per session time point (Session 1, 4, 8, and 11) with standard errors, categorized by vocalization type (delight, displeasure, neutral).

LENA pre- and post-intervention recordings assessed both adult-to-child and child language production. No changes were found in adult-to-child language measures. Among children, five out of eight child participants showed an increase in babbling post-intervention, one showed no change, and of the two who exhibited a decrease in babbling, one demonstrated a substantial increase in the number of words produced (Figure 3, Panel A). Combining babbling and words produced into a single value of “*change in expressive language by the child pre- and post-intervention”* showed there was a significant increase in expressive communication post-intervention (*M* = 9.63, *t* = 2.08, *p* = 0.038).

#### 3.2.2 Intervention sessions

Session-based LENA analyses revealed no significant effects in adult-to-child interactions. For child vocalizations, linear mixed-effects models revealed a significant increase in babbles and words across sessions [*β* = 0.013, SE = 0.006, *t*(29) = 2.21, *p* = 0.036], though only four of ten participants produced these vocalizations during play sessions. More interestingly, Figure 3 Panel B a substantial decrease in displeasure, a relatively stable number of neutral vocalizations, and an increase in delight can be observed over the course of the intervention. A composite “*non-babble vocalization*” category was used and revealed a significant increase of child vocalizations across the intervention *[β* *=* *1.56, SE* *=* *0.76, t(29)* *=* *2.07, p* *=* 0*.047.*], observed across all 10 participants.

### 3.3 Patterns of convergence across assessment tools

**Figure 4:**
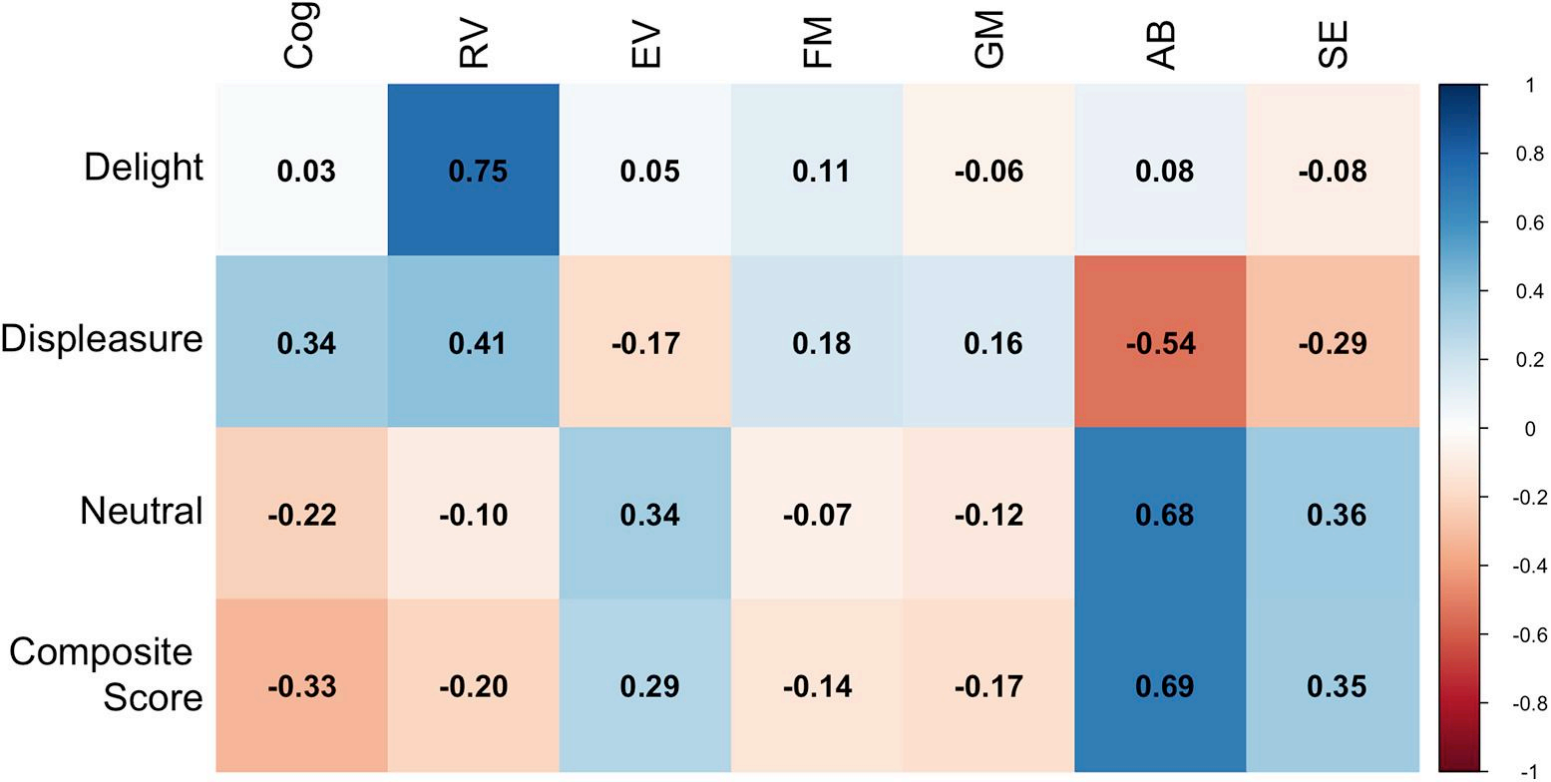
Correlation matrix showing Pearson correlation coefficients between change in vocalization rate from Session 11 and Session 1 for the following vocalization types: delight, displeasure, neutral, and composite score. These were correlated with the change in scaled scores of the BSID-IV developmental measure (post-test minus pre-test). Cog, cognitive; EV, expressive vocabulary; RV, receptive vocabulary; FM, fine moter; GM, gross moter; AB, adaptive behavior; SE, social emotional. Blue scores indicate strong positive correlations, whereas red scores indicate strong negative correlations.

Post hoc Pearson correlations explored associations between LENA-derived vocalization changes and BSID-IV scaled score improvements (Figure 4). Increases in delight vocalizations correlated strongly with receptive vocabulary gains (*r* = 0.75). Reductions in displeasure and increases in neutral vocalizations were moderately to strongly associated with improvements in adaptive behavior (*r* = −0.54 for displeasure, *r* = 0.68 for neutral, *r* = 0.69 for composite).

## 4 Discussion

The goal of this work was to establish foundational data on the developmental effects of powered mobility use in young children (12–36 months) with motor disabilities. Beyond informing researchers, this work sought to assemble a battery of cognitive, linguistic, and social measures relevant to caregivers, educators, and clinicians—tools that might encourage timely access to mobility technologies. Ten children with a diverse range of motor impairments (Table 1) participated in a 12-session in-lab intervention using the Permobil Explorer Mini, a commercially available powered mobility device designed for this population. This study investigated how providing young children with the opportunity to experience self-initiated mobility and a sense of agency, through guided learning and powered mobility, may impact their broader developmental trajectories. The domains measured included receptive and expressive language skills, cognition, gross and fine motor abilities, adaptive behavior, and socio-emotional development. To assess the intervention's impact, we utilized both pre- and post-intervention measures (e.g., BSID-IV assessments, LENA language recordings) as well as within-intervention-session metrics (e.g., LENA recordings and driving times), offering a comprehensive analysis of how the newfound agency might influence the developmental trajectories of children with motor disabilities.

The findings confirmed, based on the BSID-IV developmental scale, that our sample of young children with motor disabilities exhibited significant developmental delays beyond gross motor impairments, including expressive and receptive language, fine motor skills, cognition, adaptive behavior, and social-emotional abilities, when compared to standardized scores from age-matched typically developing peers. These results underscore the importance of providing enriched, accessible learning environments and supports that align with each child's developmental needs and strengths. Such approaches may help create more equitable opportunities for growth and participation.

Following the intervention, significant improvements were observed in raw BSID-IV scores across all developmental domains [in line with (7, 33)], except gross motor skills and expressive language. While developmental progress over a 12-week period is commonly observed in typically developing children (22), children with motor disabilities may follow different developmental timelines (35), thus these observed changes are promising.

Interestingly, participants showed gains in standardized scale scores for receptive vocabulary and cognition that exceeded what could be attributed to age-related progression during the intervention period. This suggests that even a brief intervention (6–12 weeks) with an age-appropriate powered mobility device may be associated with growth in areas beyond physical movement including cognitive, social, and communicative development. The ability for children to move autonomously, deciding when and where to move, and what to engage with, appears to have catalyzed growth in cognitive and communicative development, highlighting the importance of fostering agency in young children with motor impairments and underscoring the need for future research on this topic. The results from this study mirror previous findings of significant increases in BSID-IV scores following a 16-week powered mobility clinical trial (7).

Although improvements in the gross motor and expressive communication domains were observed on the BSID-IV from pre- to post-intervention, these did not reach statistical significance. This may be attributed to the shorter intervention period, the heterogeneity of motor disability in our study sample, or the expressive communication challenges many participants faced due to motor delays and/or the presence of medical devices that limited their ability to vocalize. As a result, standardized assessments such as the BSID-IV may underestimate emerging communication skills in children whose physical or medical constraints restrict verbal output.

In contrast, both within-intervention-session and pre/post LENA language recordings revealed a significant increase in babbles and words among children who were able to produce these vocalizations. These findings suggest that LENA recordings allow for the collection of naturalistic language data that, when manually coded, reveal subtle yet meaningful changes in expressive language development. This approach allows researchers to detect early communicative behaviors that may not yet be reflected in standardized test batteries. While manual LENA analysis is time-intensive for researchers, the data collection process remains simple and unobtrusive for caregivers and participants, requiring only that the child wear a vest containing the recording device. Collectively, these results highlight the value of LENA-enabled analysis in detecting early shifts in language development, which often align with caregivers' own perceptions of meaningful progress.

We were able to observe babble and word counts in only four of the ten participants, but we successfully quantified non-babble vocalization patterns categorized as expressions of delight, displeasure, or neutral for all ten children. Over the course of the intervention, we observed a significant increase in Delight vocalizations and a significant decrease in Displeasure vocalizations. This provides added motivation to advocate for powered mobility devices. Notably, we found significant correlations between changes in vocalization patterns and broader developmental outcomes. Specifically, increases in Delight vocalizations were significantly correlated with improvements in receptive communication skills, while an increase in Neutral or decrease in Displeasure vocalizations were linked to gains in adaptive behavior skills. These associations are exploratory and do not establish causality, but they suggest that enhanced agency, afforded by powered mobility, may play a role in fostering developmental progress across multiple domains.

Building on the positive impact observed in vocalization patterns, particularly those reflecting emotion (Delight and Displeasure), it is important to highlight that this intervention was rewarding for both the children and their caregivers. Clinically, both we as researchers, and the caregivers, frequently noted that the children became eager to engage with the Explorer Mini, relished the social interactions with researchers and caregivers, and enthusiastically embraced the challenges presented by the toys and the powered mobility device. Some children mastered complex path navigation, and others focused more on goal-directed driving attempts, nonetheless, caregivers across the board reported noticeable increases in their children's joy and development.

Anecdotally, caregivers also drew our attention to the profound importance of agency in their children's well-being. One caregiver reflected, “*It's maybe one of the first things that she's been able to do that shows that she has some agency… I feel like she got some joy out of it, so that's been really nice to see. That she can bring joy to herself.”* (Caregiver, P1). Another caretaker remarked on the cognitive impact of mobility, stating, “*I think that this mobility would broaden him cognitively, you know. It's instead of thinking of himself, like, in that infancy stage where ‘the adults take you places,’ it's for him to bridge that gap to, ‘I take me places!’”* (Caregiver, P5). These reflections underscore how mobility interventions can foster not only joy but also critical cognitive and emotional growth by enabling children to experience autonomy in meaningful ways.

### 4.1 Limitations

This study has several limitations. First, the sample size was small (*N* = 10) and heterogeneous, limiting statistical power and generalizability. Second, the absence of a control group precludes causal inference; observed changes may reflect natural developmental progression or other unmeasured factors. Third, the analyses were exploratory, and multiple comparisons increase the risk of Type I error. Finally, the English-centric nature of LENA analysis may have limited the accuracy of language measures for participants from multilingual homes.

### 4.2 Strengths

Despite these limitations, the study has notable strengths. The integration of powered mobility intervention with both standardized developmental assessments and naturalistic language sampling (LENA) is innovative and provides a more comprehensive view of developmental change. The use of both pre/post and within-session data allowed for the detection of subtle, session-by-session changes in communicative behavior. The study also included a diverse sample of children with a range of motor impairments, enhancing the practical relevance of the findings.

## 5 Conclusion

This exploratory study provides preliminary evidence that powered mobility interventions may support developmental progress in young children with motor disabilities, particularly in cognitive, communicative, and socio-emotional domains. Studies such as this are essential for laying a foundational framework to highlight the comprehensive developmental implications of what may initially appear to be isolated delays, such as motor impairments, in young children. The results highlight the potential of mobility technologies to foster agency and engagement, and underscore the need for more rigorous research to guide clinical practice and policy.

Ultimately, this study reinforces the deep and dynamic interconnectedness of motor and communication development in early childhood. By enabling self-directed mobility, we did not merely facilitate physical movement, we opened new pathways for exploration, engagement, and expression. The observed gains in cognitive, communicative, and socio-emotional domains underscore that mobility is not an isolated motor skill, but a catalyst for broader developmental growth, and call for a more integrated approach to early intervention. An approach that recognizes how empowering children with motor disabilities through mobility can ignite cascading developmental progress.

## Data Availability

The raw data supporting the conclusions of this article will be made available by the authors, without undue reservation.

## References

[1] Besio S Amelina N. Play in children with physical impairment. In: Play Development in Children with Disabilties (Originally published 2016, p. 120–36). De Gruyter Open. (2017). 10.1515/9783110522143-011

[2] Iverson JM. Developing language in a developing body, revisited: the cascading effects of motor development on the acquisition of language. WIRES Cogn Sci. (2022) 13(6):e1626. 10.1002/wcs.1626 PMC12333486 36165333

[3] Anderson DI Campos JJ Witherington DC Dahl A Rivera M He M The role of locomotion in psychological development. Front Psychol. (2013) 4:440. 10.3389/fpsyg.2013.00440 23888146 PMC3719016

[4] Bray N Kolehmainen N McAnuff J Tanner L Tuersley L Beyer F Powered mobility interventions for very young children with mobility limitations to aid participation and positive development: the EMPoWER evidence synthesis. Health Technol Assess. (2020) 24(50):1. 10.3310/hta24500 33078704 PMC7681349

[5] Adolph KE Cole WG Komati M Garciaguirre JS Badaly D Lingeman JM How do you learn to walk? Thousands of steps and dozens of falls per day. Psychol Sci. (2012) 23(11):1387–94. 10.1177/0956797612446346 23085640 PMC3591461

[6] Iverson JM. Developing language in a developing body: the relationship between motor development and language development. J Child Lang. (2010) 37(2):229–61. 10.1017/S0305000909990432 20096145 PMC2833284

[7] Feldner HA Logan SW Otieno S Fragomeni A Kono C Riordan K Short-term powered mobility intervention is associated with improvements in development and participation for young children with cerebral palsy: a randomized clinical trial. Phys Ther. (2025) 105(1):pzae152. 10.1093/ptj/pzae152 39450982

[8] Hospodar CM Feldner HA Logan SW. Active mobility, active participation: a systematic review of modified ride-on car use by children with disabilities. Disabil Rehabil. (2023) 18(6):974–88. 10.1080/17483107.2021.1963330 PMC9328769 34435924

[9] Lobo MA Harbourne RT Dusing SC McCoy SW. Grounding early intervention: physical therapy cannot just be about motor skills anymore. Phys Ther. (2013) 93(1):94–103. 10.2522/ptj.20120158 23001524 PMC3538987

[10] Ragonesi CB Chen X Agrawal S Galloway JC. Power mobility and socialization in preschool: a case study of a child with cerebral palsy. Pediatr Phys Ther. (2010) 22(3):322–9. 10.1097/PEP.0b013e3181eab240 20699785

[11] Permobil. Permobil Explorer Mini (2022). Available online at: https://www.permobil.com/en-us/products/powerwheelchairs/permobil-explorer-mini (Accessed June, 2024).

[12] Logan SW Feldner HA Bogart KR Catena MA Hospodar CM Raja Vora J Perceived barriers of modified ride-on car use of young children with disabilities: a content analysis. Pediatr Phys Ther. (2020) 32(2):129–35. 10.1097/PEP.0000000000000690 32150029 PMC7546953

[13] Logan SW Feldner HA Bogart KR Catena MA Hospodar CM Raja Vora J Perceived barriers before and after a 3-month period of modified ride-on car use. Pediatr Phys Ther. (2020) 32(3):243–8. 10.1097/PEP.0000000000000711 32604369 PMC7561053

[14] Sloane BM Kenyon LK Logan SW Feldner HA. Caregiver perspectives on powered mobility devices and participation for children with cerebral palsy in gross motor function classification system level V. Dev Med Child Neurol. (2024) 66(3):333–43. 10.1111/dmcn.15718 37515376

[15] Logan SW Sloane BM Kenyon LK Feldner HA. Powered mobility device use and developmental change of young children with cerebral palsy. Behav Sci. (2023) 13(5):399. 10.3390/bs13050399 37232636 PMC10215286

[16] Plummer T Logan SW Morress C. Explorer Mini: infants’ initial experience with a novel pediatric powered mobility device. Phys Occup Ther Pediatr. (2021) 41(2):192–208. 10.1080/01942638.2020.1819935 33019827

[17] LUCI. Modern Mobility (2020). Available online at: https://luci.com (Accessed June, 2024).

[18] Ingraham KA Zaino NL Feddema C Hoffman ME Gijbels L Sinclair A Quantifying joystick interactions and movement patterns of toddlers with disabilities using powered mobility with an instrumented explorer Mini. IEEE Trans Neural Syst Rehabil Eng. (2025) 33:431–40. 10.1109/TNSRE.2025.3528454 40031044

[19] Zaino NL Ingraham KA Hoffman ME Feldner HA Steele KM. Quantifying toddler exploration in different postures with powered mobility. Assist Technol. (2025) 37(2):93–101. 10.1080/10400435.2024.2400463 39401285

[20] Nilsson L Durkin J. Assessment of learning powered mobility use—applying grounded theory to occupational performance. J Rehabil Res Dev. (2014) 51(6):963–74. 10.1682/JRRD.2013.11.0237 25357100

[21] Feldner H Plummer T Hendry A. A guideline for introducing powered mobility to infants and toddlers. Permobil. (2022). Available online at: https://permobilwebcdn.azureedge.net/media/stwou5go/a-guideline-for-introducing-powered-mobility-to-infants-and-toddlers_v0122.pdf (Accessed January, 2023).

[22] Bayley N Aylward GP. Bayley-4: Scales of Infant and Toddler Development. Technical Manual. 4th edn. Hoboken NJ: Pearson Publications (2019).

[23] LENA. LENA™ Pro Version 3.4.0. Boulder, CO: LENA (2015). Available online at: http://www.lenafoundation.org/ (Accessed June, 2024).

[24] Ferjan Ramírez N Hippe DS Kuhl PK. Comparing automatic and manual measures of parent–infant conversational turns: a word of caution. Child Dev. (2021) 92(2):672–81. 10.1111/cdev.13495 33421100 PMC8048438

[25] Ferjan Ramírez N Lytle SR Fish M Kuhl PK. Parent coaching at 6 and 10 months improves language outcomes at 14 months: a randomized controlled trial. Dev Sci. (2019) 22(3):e12762. 10.1111/desc.12762 30318708

[26] Ramírez-Esparza N García-Sierra A Kuhl PK. The impact of early social interactions on later language development in Spanish-English bilingual infants. Child Dev. (2017) 88(4):1216–34. 10.1111/cdev.12648 27759883

[27] Solomon O. Rethinking baby talk. In: Duranti A Ochs E Schieffelin BB, editors. The Handbook of Language Socialization. Oxford: Blackwell Publishing Ltd (2011). p. 121–49. 10.1002/9781444342901.ch5

[28] Grieser DL Kuhl PK. Maternal speech to infants in a tonal language: support for universal prosodic features in motherese. Dev Psychol. (1988) 24:14–20. 10.1037/0012-1649.24.1.14

[29] Ferjan Ramírez N. What do parents really think? Knowledge, beliefs, and self-awareness of parentese in relation to its use in daylong recordings. First Lang. (2024) 44(1):3–22. 10.1177/01427237231216010

[30] Paul R Fuerst Y Ramsay G Chawarska K Klin A. Out of the mouths of babes: vocal production in infant siblings of children with ASD. J Child Psychol Psychiatry. (2011) 52(5):588–98. 10.1111/j.1469-7610.2010.02332.x 21039489 PMC3078949

[31] Schoen E Paul R Chawarska K. Phonology and vocal behavior in toddlers with autism spectrum disorders. Autism Res. (2011) 4(3):177–88. 10.1002/aur.183 21308998 PMC3110574

[32] Sheinkopf SJ Mundy P Oller DK Steffens M. Vocal atypicalities of preverbal autistic children. J Autism Dev Disord. (2000) 30:345–54. 10.1023/A:1005531501155 11039860

[33] Jones MA McEwen IR Neas BR. Effects of power wheelchairs on the development and function of young children with severe motor impairments. Pediatr Phys Ther. (2012) 24(2):131–40. 10.1097/PEP.0b013e31824c5fdc 22466379

[34] Maitre NL Jeanvoine A Yoder PJ Key AP Slaughter JC Carey H Kinematic and somatosensory gains in infants with cerebral palsy after a multi-component upper-extremity intervention: a randomized controlled trial. Brain Topogr. (2020) 33(6):751–66. 10.1007/s10548-020-00790-5 32748303

[35] Applegate A Spicer CM Frontera W. Low Birth Weight Babies and Disability (2024). 39088674

[36] Bates D Mächler M Bolker B Walker S. Fitting linear mixed-effects models using lme4. J Stat Softw. (2015) 67(1):1–48. 10.18637/jss.v067.i01

